# Supplementary material for: Strain-driven growth of ultra-long two-dimensional nano-channels
Source: Nat Commun. 2020 Feb 7;11:772. doi: 10.1038/s41467-020-14521-8 (PMC7005715; doi:10.1038/s41467-020-14521-8)
Supplement: Supplementary file 1 — Supplementary Information [file 41467_2020_14521_MOESM1_ESM.pdf]

Supplementary Information for

Strain-driven growth of ultra-long two-dimensional nano-channels

Chao Zhu[1,2†], Maolin Yu[3†], Jiadong Zhou[1†], Yongmin He[1], Qingsheng Zeng[1], Ya Deng[1], Shasha Guo[1], Mingquan Xu[2], Jinan Shi[2], Wu Zhou[2], Litao Sun[4], Lin Wang[5], Zhili Hu[3], Zhuhua Zhang[3*], Wanlin Guo[3*] & Zheng Liu[1,6,7*]

[1]School of Materials Science and Engineering, Nanyang Technological University, Singapore 639798, Singapore.

[2]School of Physical Sciences, CAS Key Laboratory of Vacuum Physics, University of Chinese Academy of Sciences, Beijing 100049, China.

[3]State Key Laboratory of Mechanics and Control of Mechanical Structures, Key Laboratory for Intelligent Nano Materials and Devices of Ministry of Education, and Institute of Nanoscience, Nanjing University of Aeronautics and Astronautics, Nanjing 210016, China

[4]SEU-FEI Nano-Pico Center, Key Laboratory of MEMS of Ministry of Education, Collaborative Innovation Center for Micro/Nano Fabrication, Device and System, Southeast University, Nanjing 210096, P. R. China.

[5]Key Laboratory of Flexible Electronics & Institute of Advanced Materials, Jiangsu National Synergetic Innovation Center for Advanced Material, Nanjing Tech University, 30 South Puzhu Road, Nanjing 211816, China.

[6]Environmental Chemistry and Materials Centre, Nanyang Environment and Water Research Institute, Singapore, Singapore.

[7]CINTRA CNRS/NTU/THALES, UMI 3288, Research Techno Plaza, 50 Nanyang Drive, Border X Block, Level 6, Singapore 637553, Singapore.

[†]These authors contributed equally to this work

[*]email: chuwazhang@nuaa.edu.cn; wlguo@nuaa.edu.cn; z.liu@ntu.edu.sg

Supplementary Figures

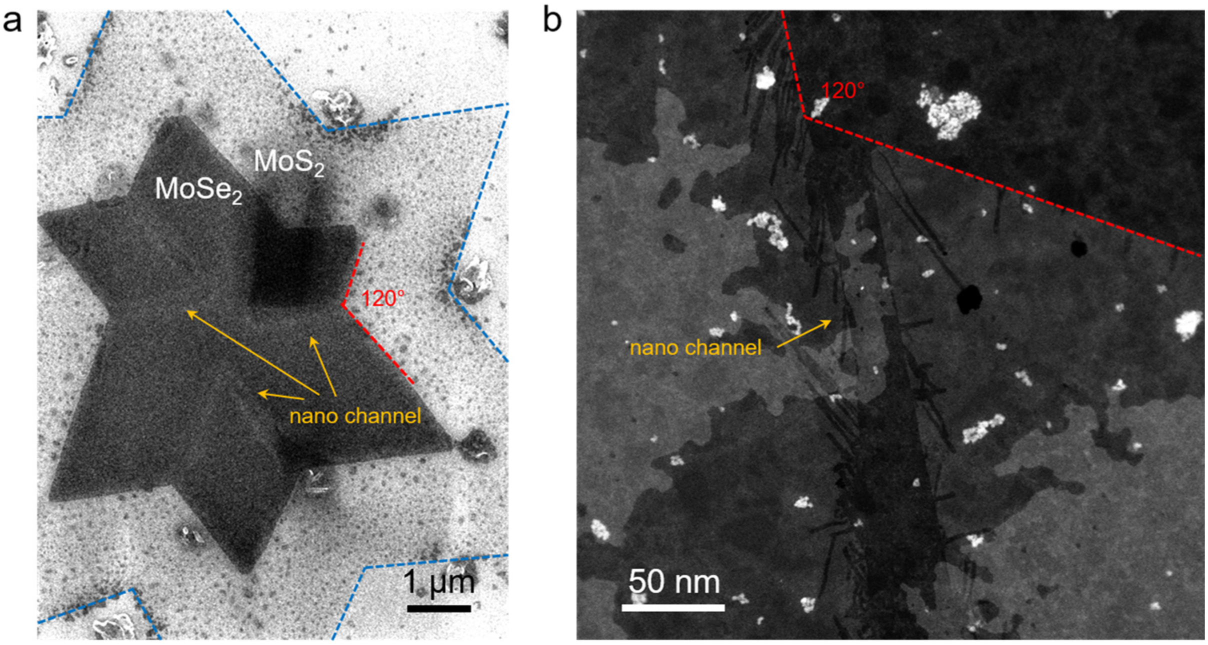

Supplementary Figure 1. Locations of MoS_2 nano-channels in $MoSe_2$ matrix. a, SEM image of a MoS_2-$MoSe_2$ heterostructure flake. The epitaxial MoS_2 has the same 6-point star shape (edges are labeled by the dashed blue lines) with the inner $MoSe_2$ monolayer. MoS_2 nano-channels, brighter contrast lines as indicated by the arrows, locate between neighbored corners and divide the $MoSe_2$ matrix into several parallelogram pieces. **b**, Large scale ADF-STEM image of the heterojunction area showing that the nano-channel origins exactly from the vertexes of obtuse angles (120º) between neighbored corners.

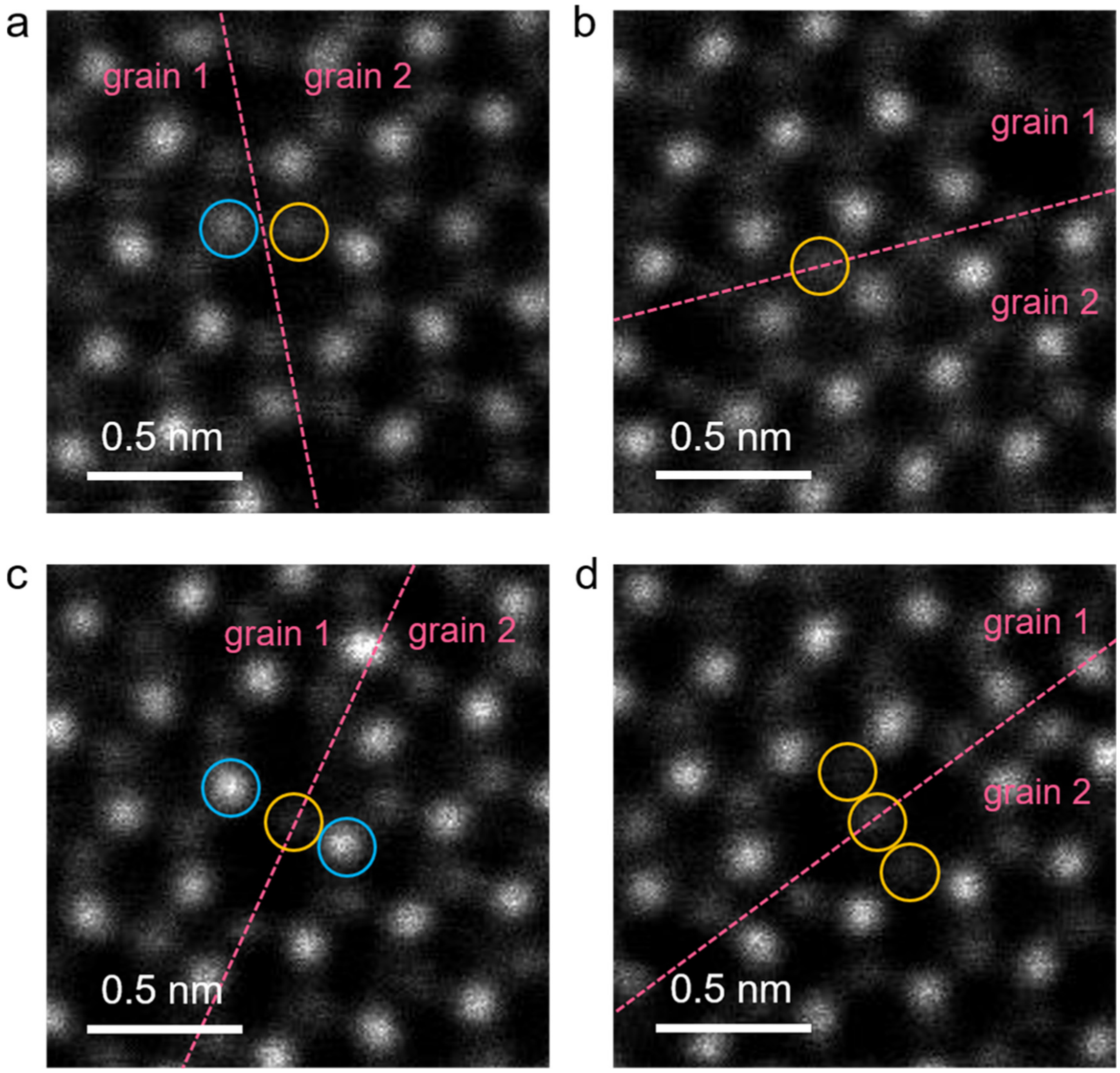

Supplementary Figure 2. Four joint types of 60º GBs. a, Type I: Mo atoms from one grain bond with S atoms from the other. In this type, Mo and S atoms at the GB maintain 6-fold and 3-fold coordination respectively, the same as those in defectless MoS_2 hexagonal lattice. **b**, Type II: two grains share the same S atoms with 4-fold coordination. **c**, Type III: two grains share the same 2-fold coordinative S atoms that bond with Mo atoms in each grain. **d**, Type IV: two grain share same 2-fold coordinative S atoms that bond with S atoms in each grain. Here, blue and yellow circles represent Mo and S atoms. In our observation, 60º GBs are mainly composed of the former three joint ways, leading to the successive 4|8 rings. The density of type IV is very low, probably due to the energetically unfavorable arrangement of S atoms in this case.

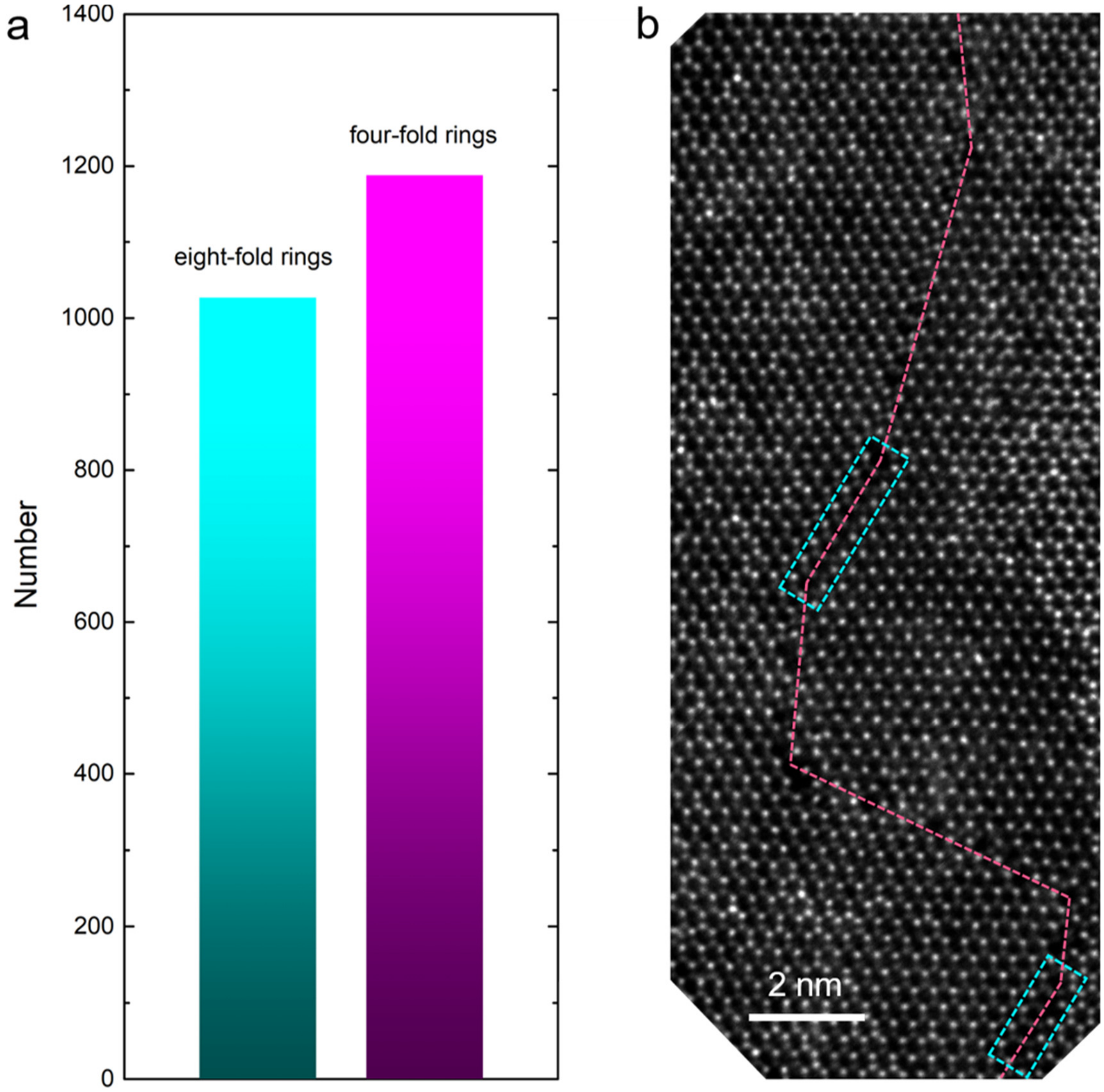

Supplementary Figure 3. Statistics of four- and eight-fold rings. a, Histogram of the number of four- and eight-fold rings along 1 μm-length 60º GBs, yielding a ratio of 1:1.16. **b**, ADF-STEM image shows the short chains of parallel eight-fold rings (highlighted by the rectangles).

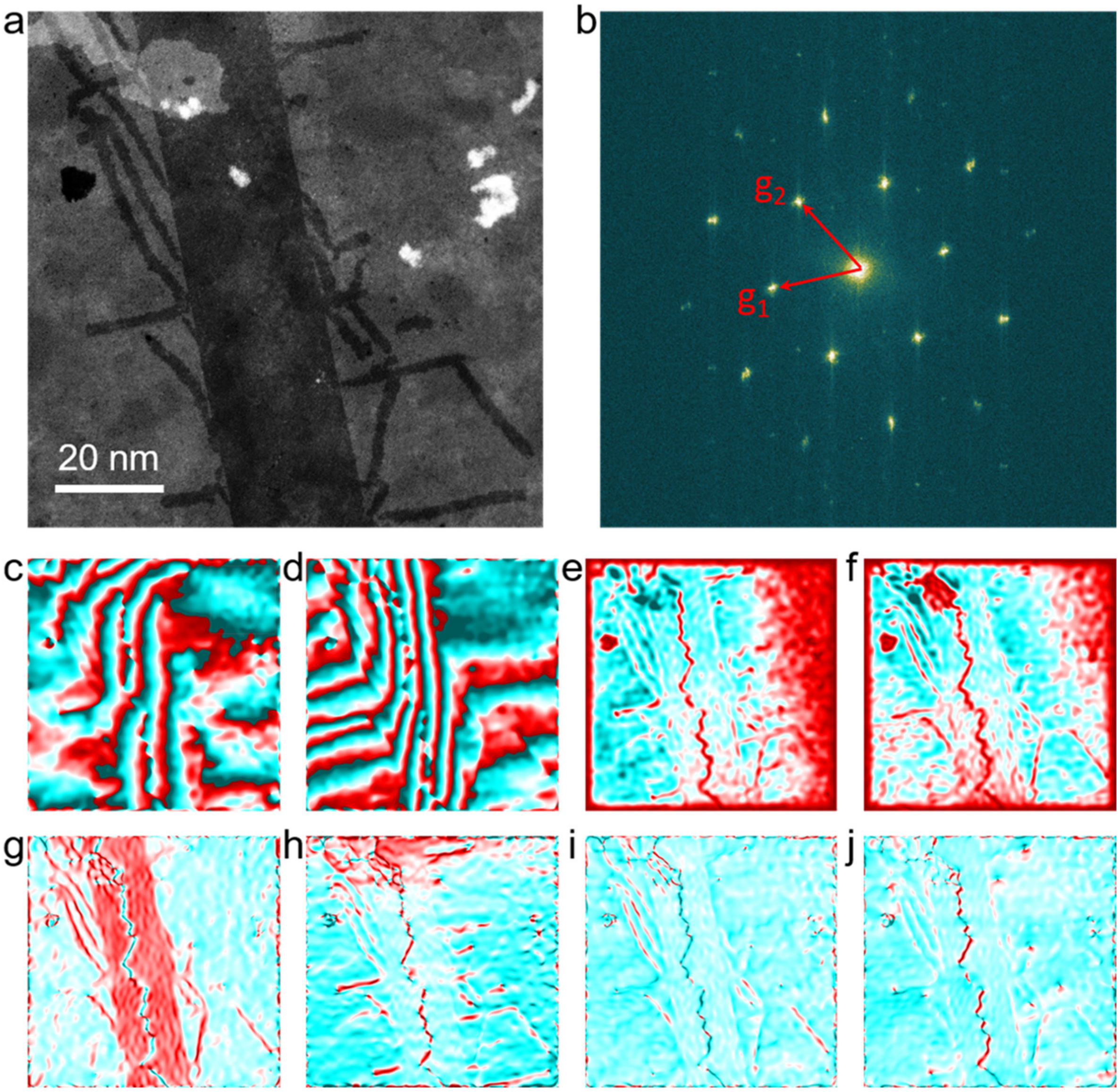

Supplementary Figure 4. Geometric phase analysis. **a**, Low-magnification ADF-STEM image of a MoS_2 channel embedded in $MoSe_2$ matrix. **b**, FFT image of **a**, where $\mathbf{g_1}$ and $\mathbf{g_2}$ are selected as the Bragg reflections for phase and strain calculation. **c** and **d**, Phase images corresponding to $\mathbf{g_1}$ and $\mathbf{g_2}$. **e** and **f**, Scale of reciprocal lattice $\mathbf{g_1}$ and $\mathbf{g_2}$. **g-i**, Strain maps (ε_{xx}, ε_{yy}, ε_{xy}) of **a**. **j**, Rotation map of **a**.

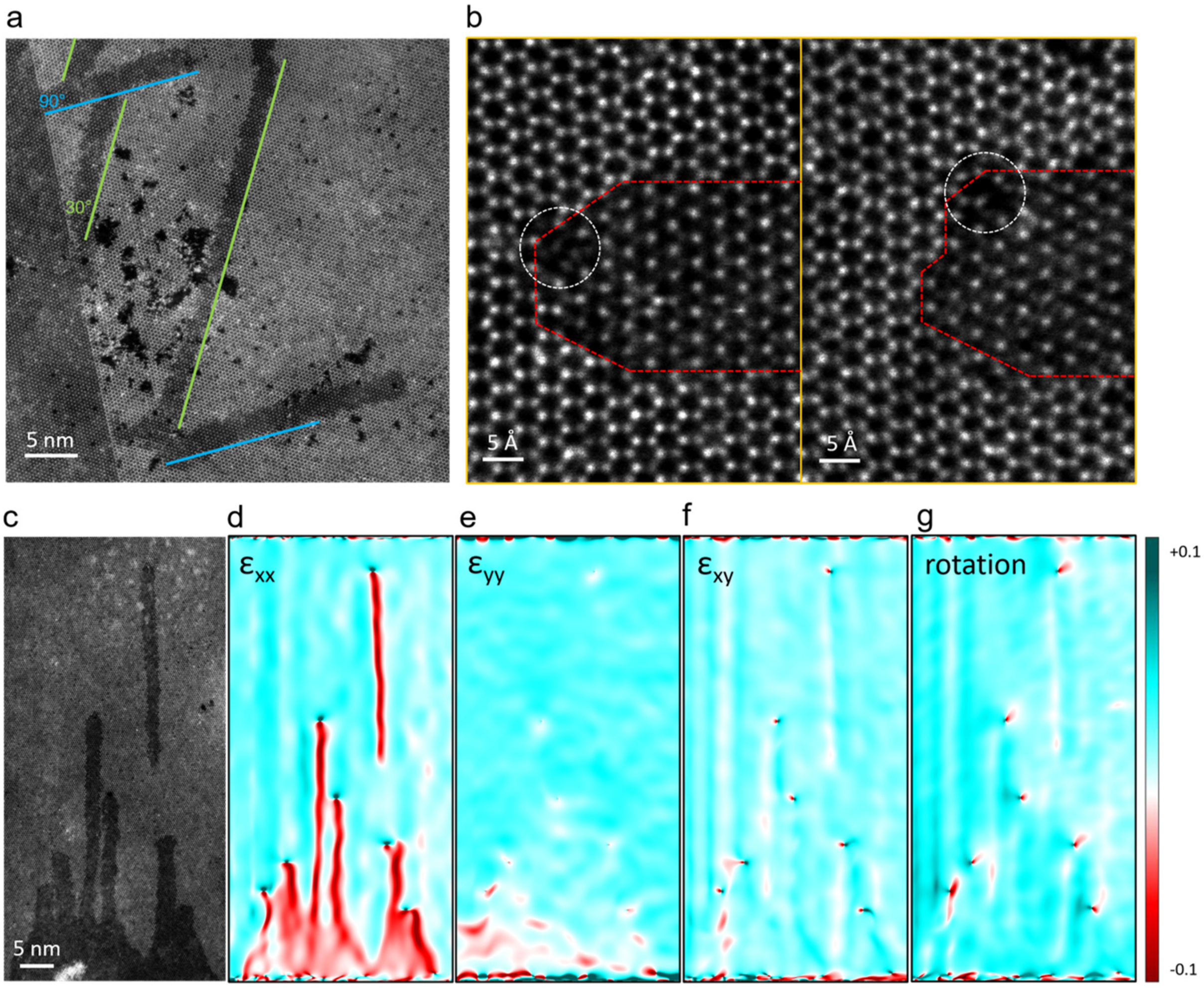

Supplementary Figure 5. Structure and GPA analysis of 2-nm-wide quantum wells. a, ADF-STEM image showing the growth direction of branched quantum wells: 30º (indicated by green lines) and 90º (indicated by blue lines) with respect to MoS_2 channels, consistent with our observation that straight channels grow along zigzag direction while quantum wells grow along the armchair direction of the crystal lattice. **b**, Atomic resolution ADF-STEM images showing the structure of quantum well tips. Point defects (dislocations) can be found at the tip of each quantum well (highlighted by the circles). In our observation, these defects could be missing atoms, replaced atoms or multi-fold rings such as 4- and 8- fold rings, not just 5|7 rings in previous literature. **c**-**g**, ADF-STEM image of quantum wells (**c**) and corresponding strain (**d**-**f**) and rotation maps (**g**). There are some similarities between quantum wells and nano-channels: they both have compressive ε_{xx} strain but uniform ε_{yy} strain compared with $MoSe_2$ matrix (see discussion in the main text). However, in shear strain and rotation maps, the point defect nature of quantum wells can be clearly distinguished from the line defect nature of nano-channels.

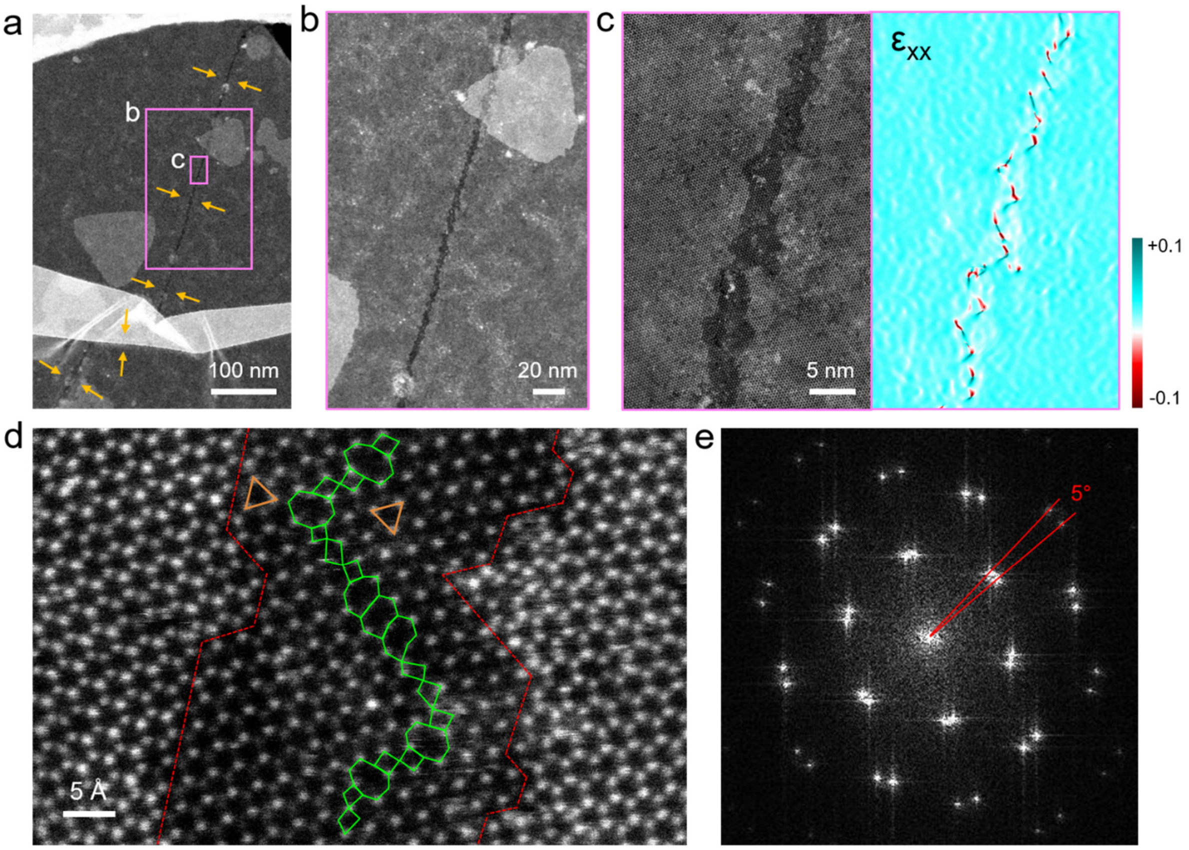

Supplementary Figure 6. MoS_2 nano-channel without quantum wells obtained at high temperature. **a**, Large field of view ADF-STEM image of a nano-channel without quantum wells. **b**, Enlarged ADF-STEM image of large rectangular region in **a**. **c**, Enlarged ADF-STEM image and corresponding strain map of the small rectangular region in **a**. **d**, High-resolution ADF-STEM image of this nano-channel showing its atomic structure. **e**, Fast Fourier transformation of **c**.

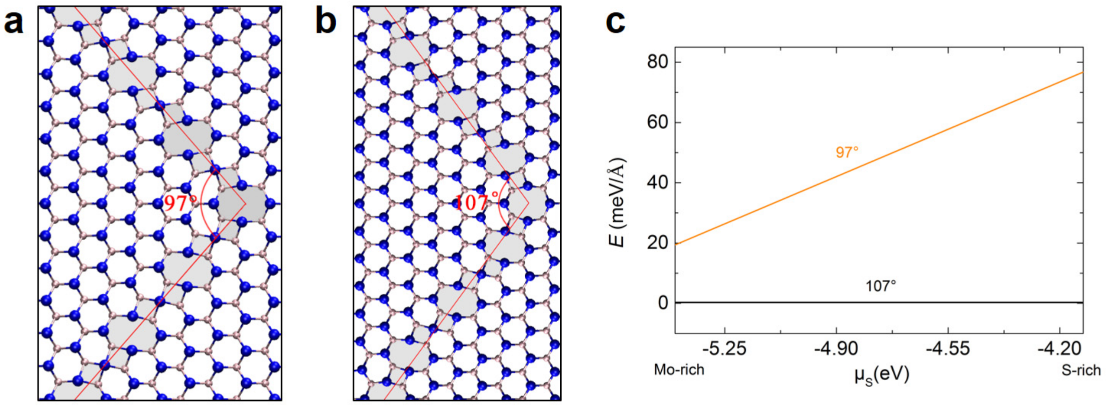

Supplementary Figure 7. Atomic structures of folded GBs. **a**, The one composed of solely of the 8|4|4|8 dislocations. **b**, The GB composed of mixed 8|4 + 8|4|4|8 dislocations. **c**, Energies of 97º GB relative to 107º GB as functions of the chemical potential of sulfur in the range −5.4 eV < μ_S < -4.1 eV.

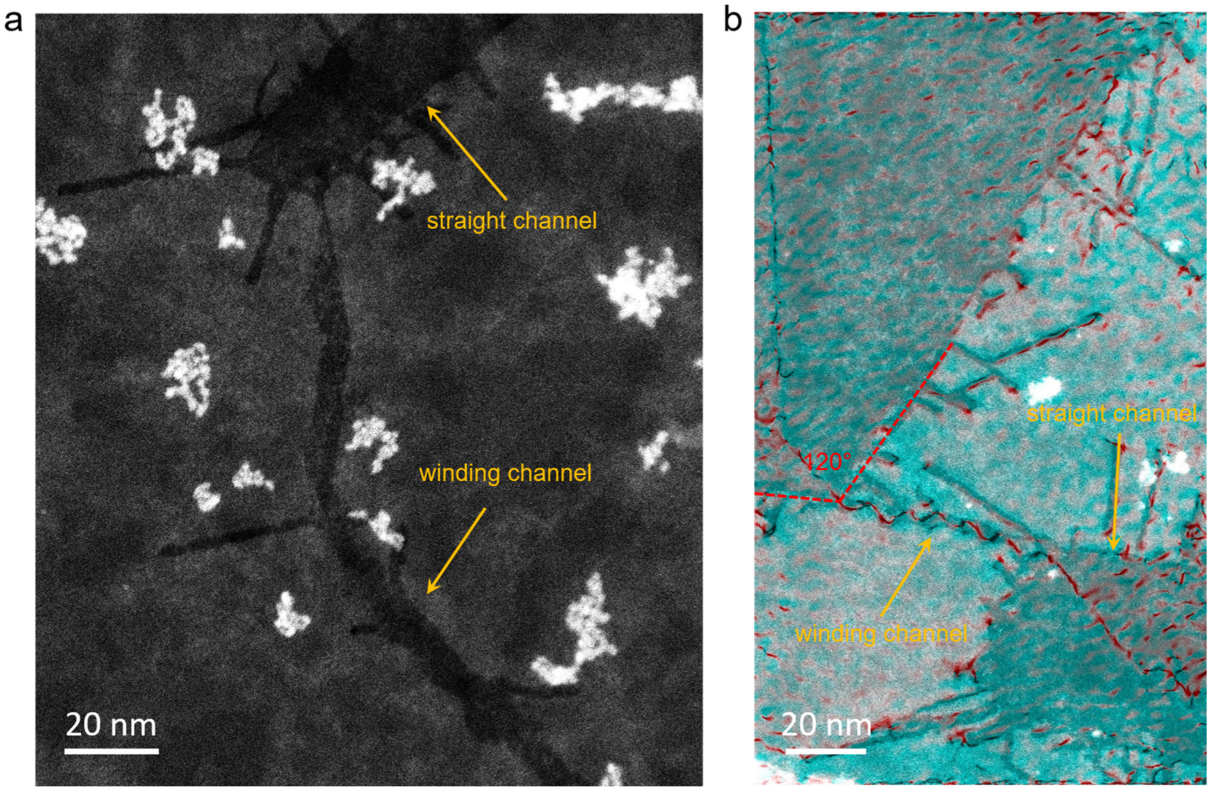

Supplementary Figure 8. Connected straight and winding channels. a, ADF-STEM image presenting the connected straight channels (20 nm in width) and winding channels (2-10 nm in width). **b**, Superimposed image of the connected channels and the corresponding rotation map. The inner zigzag 60º GB seamlessly extends from the winding channel to straight one, suggesting the consistency of them.

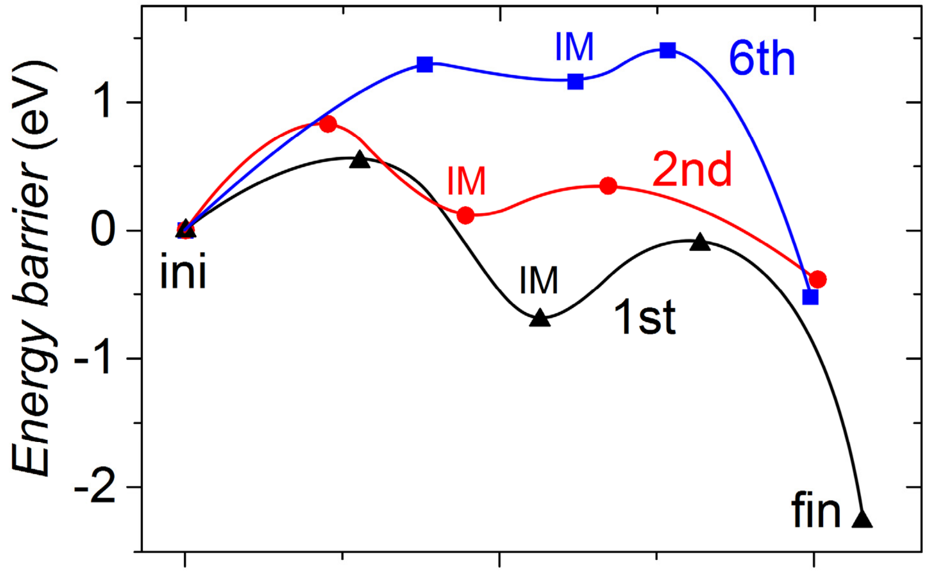

Supplementary Figure 9. Calculated minimum energy pathways energy for taking the 1st, 2nd and 6th Se atom as the substitution sites for the first S atom (see Fig. 5b). The intermediate state is marked by IM in each energy pathway.

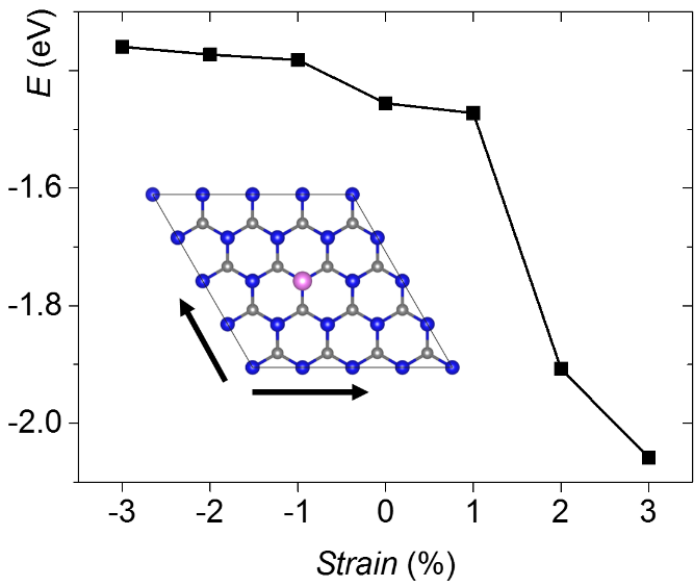

Supplementary Figure 10. Evolution of absorption energy of H atoms on a $MoSe_2$ sheet against applied lattice strain. Here we use the absorption energy of H atoms to reflect the correlation between the chemical reactivity and strain while excluding the effect of defects. We calculate the variation of absorption energy of H atoms on a perfect $MoSe_2$ sheet. The absorption energy of H atoms drops by ~0.6 eV when a moderate 3% tensile strain is applied. In comparison, the lattice strain near the GB can be up to 4% and therefore is sufficient to regulate the chemical activity thereof. We thus conclude that the chemical reactivity around the GBs is dominated by the strain. The intrinsic chemical activity of 4|8 dislocation plays a secondary role. The S substitution reaction is mainly attributed to the strain-driven mechanism as the reaction sequences also perfectly imprinted the strain patterns derived from the GBs. The inset presents the atomic structure (blue, grey and pink circles: Se, Mo and H atoms). The black arrow denotes the direction of strain.

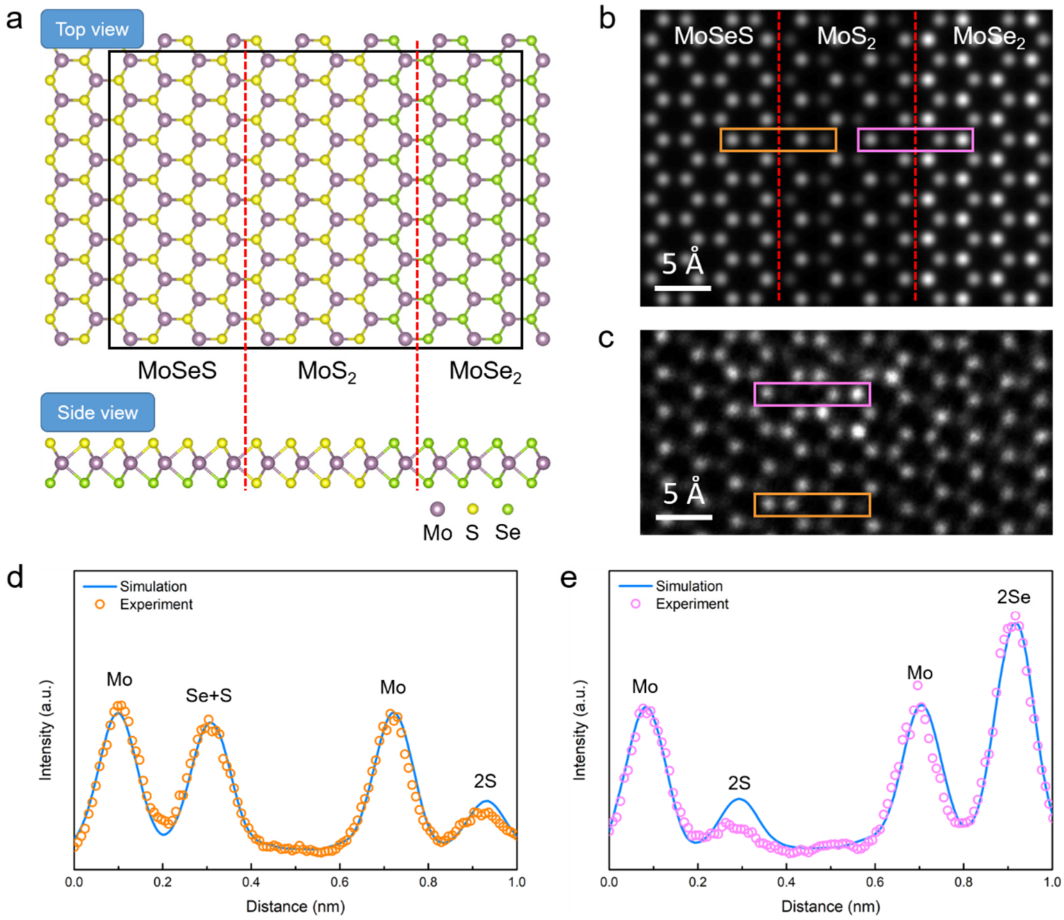

Supplementary Figure 11. Determination of SeS, SS and SeSe atom column through ADF-STEM images. **a**, Atomic model of lateral hetero-structure of monolayer MoSeS-MoS_2-$MoSe_2$. **b**, Simulated ADF-STEM image corresponding to the rectangular region of **a**. **c**, Experimental ADF-STEM image of some doped region in MoS_2 channel. **d**, Line intensity profile of simulated (orange rectangle in **b**) and experimental (orange rectangle in **c**) atom chains of Mo-SeS-Mo-2S. **e**, Line intensity profile of simulated (pink rectangle in **b**) and experimental (pink rectangle in **c**) atom chains of Mo-2S-Mo-2Se.

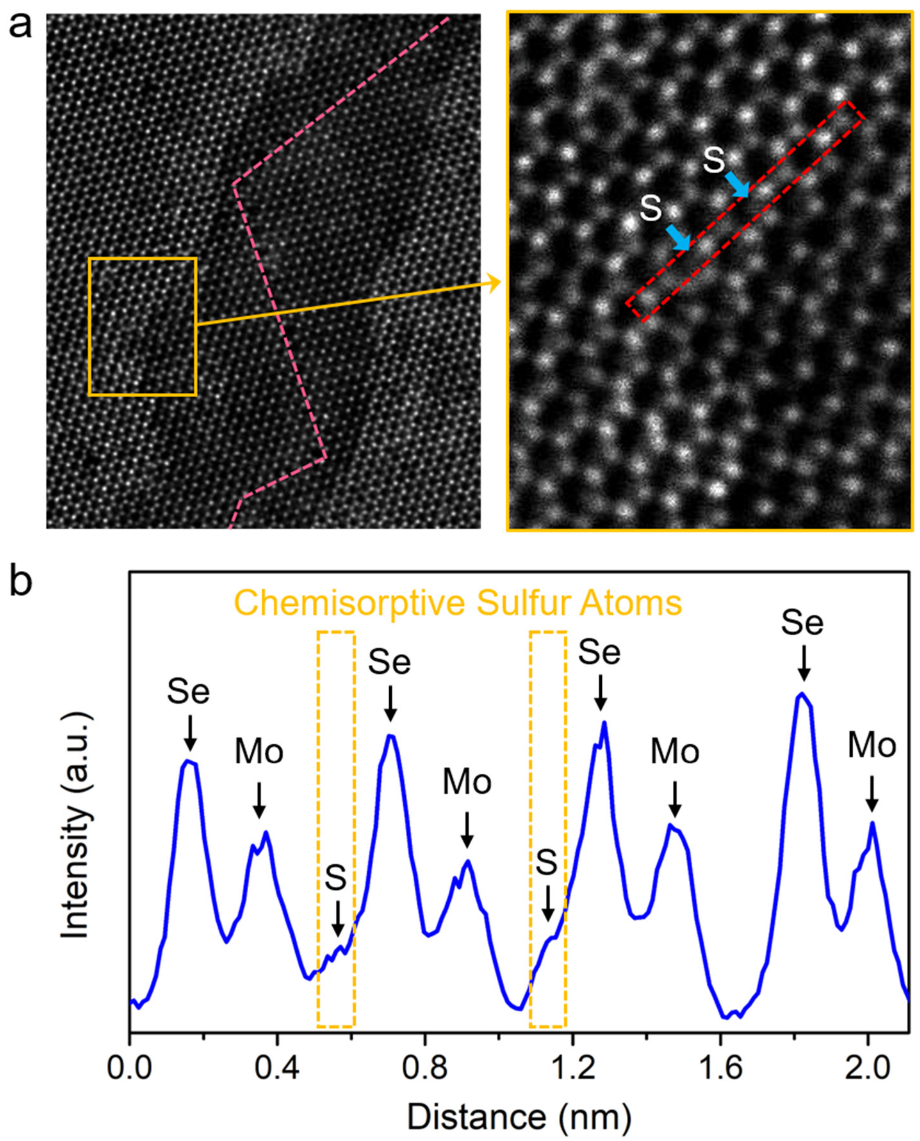

Supplementary Figure 12. Chemisorptive sulfur atoms at the interface. a, ADF-STEM image of the MoS_2 channel and the enlarged image of the $MoSe_2$-MoS_2 interface. **b**, Line intensity profile of the rectangular region in **a**, from which some chemisorptive sulfur atoms can be found at the interface, indicating the consistency of theory and experiments.

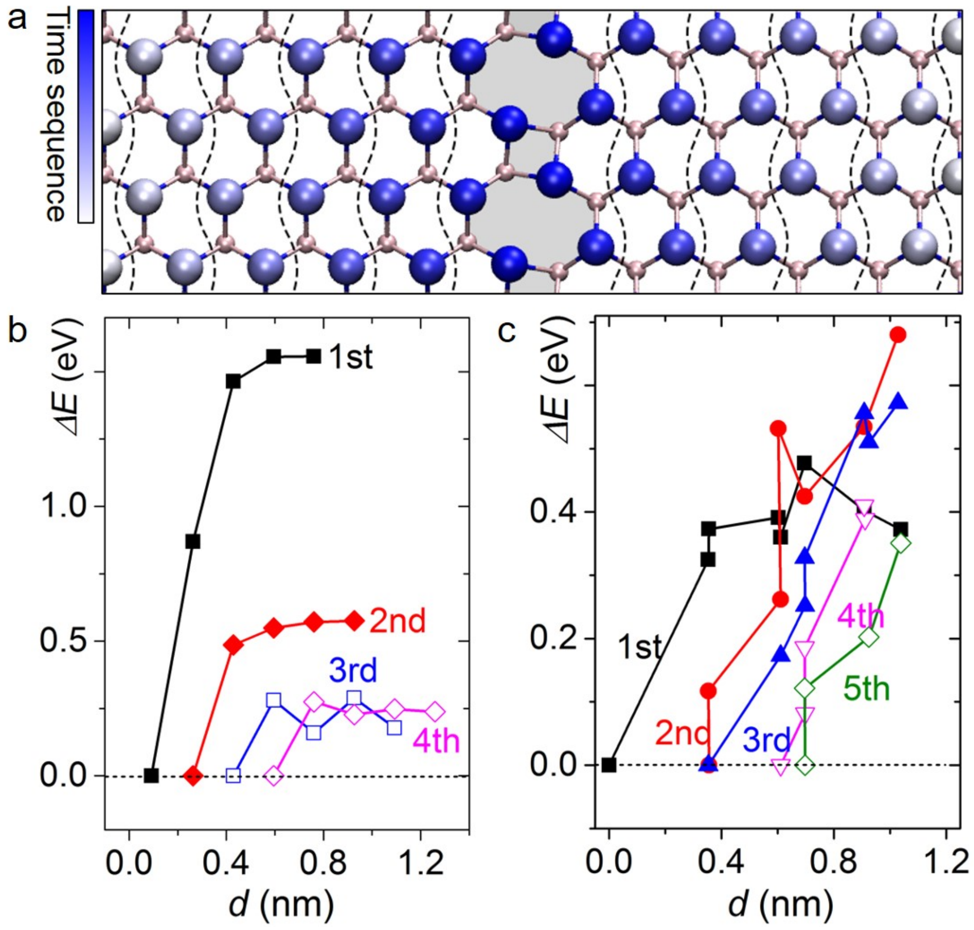

Supplementary Figure 13. a, Contour plot of substitution sequence (from blue to light grey) around the 8|4|8|4 GB. **b** and **c**, Calculated relative energies Δ*E* for the intermediate state as a function of *d* (the distance from the 8|4|8|4 GB or 5|7 dislocation, respectively) along the optimal substitution pathway.

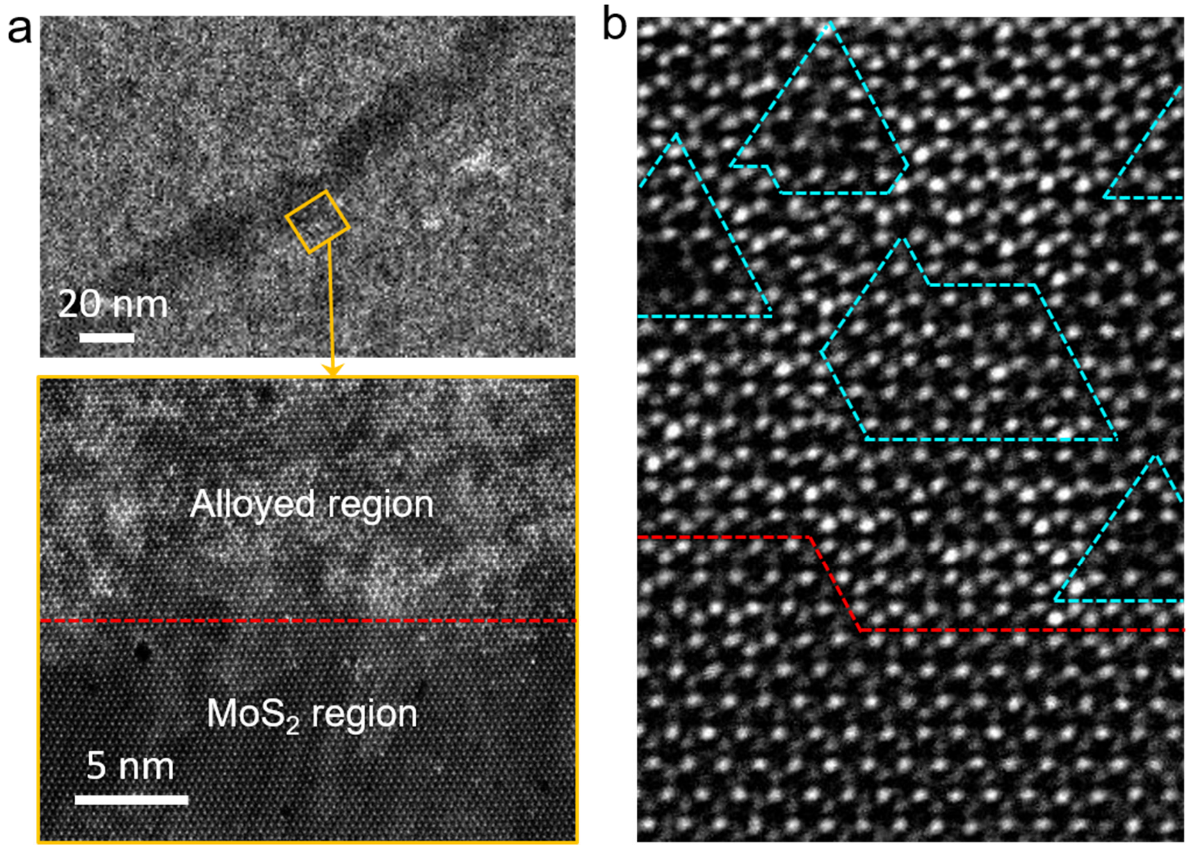

Supplementary Figure 14. Strain effect during substitution growth. In order to prove the strain-driven growth mechanism, we create some point defects (Mo and Se vacancies) with low strain through 5w Ar plasma treatment for 10 s. After that, MoS_2 growth was carried out. **a**, ADF-STEM images showing that the GBs based MoS_2 channel still can be obtained, whereas the original $MoSe_2$ region has become $MoSe_2$-MoS_2 alloy. **b**, Atomic-resolution ADF-STEM image of the interface area. The alloyed region actually consists of many MoS_2 islands (highlighted by the cyan dashed lines) with the size smaller than 2 nm, which are generated from the introduced point defects. In comparison, the channel can grow to 20 nm wide under the same condition, implying that the substitution growth occurs more easily around GBs with high strain than around vacancies with low strain.

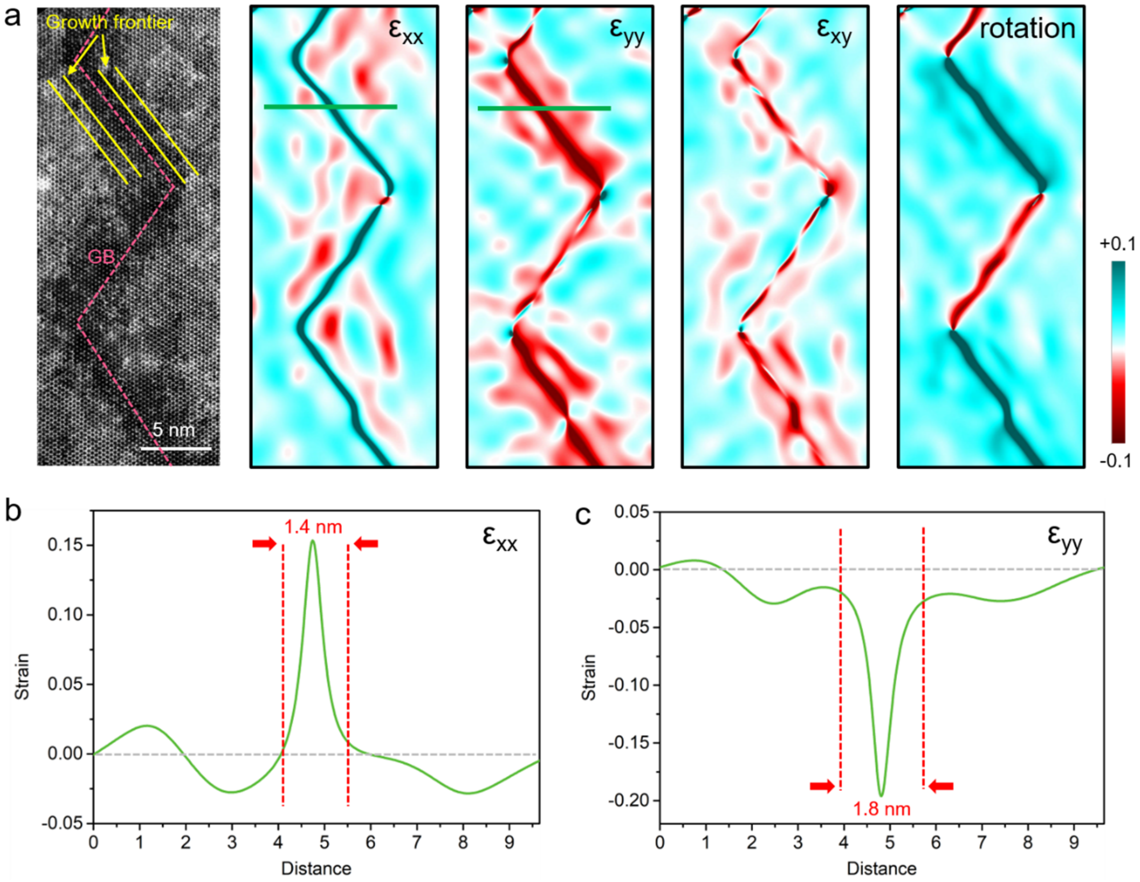

Supplementary Figure 15. Strain analysis of the growth intermediate state of MoS_2 nano-channels. **a**, ADF-STEM image of a partly-formed MoS_2 nano-channel embedded in $MoSe_2$, and the corresponding strain maps. The GB is noted with dashed lines and the growth frontier is highlighted with yellow solid lines. **b**, The line profile from the green line region in ε_{xx} strain map. **c,** The line profile from the green line region in ε_{yy} strain map. It can be confirmed from this figure that the driving force diminishes as the growth frontier moves away from the GB, and more specifically the driving force can be completely ignored when the growth frontier is 1 nm away from the GB.

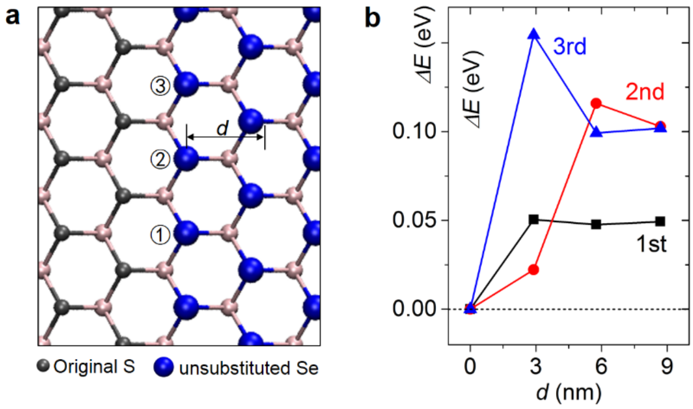

Supplementary Figure 16. a, Atomic structure of the perfect MoS_2-$MoSe_2$ interface. The numbers 1~3 denote the sequence of S substitution. *d* denotes the distance of reaction sites with respect to the interface line. **b**, Calculated relative energies ΔE for the intermediate state in **a** as a function of *d* for the 1~3 S atoms along the optimal substitution pathway.

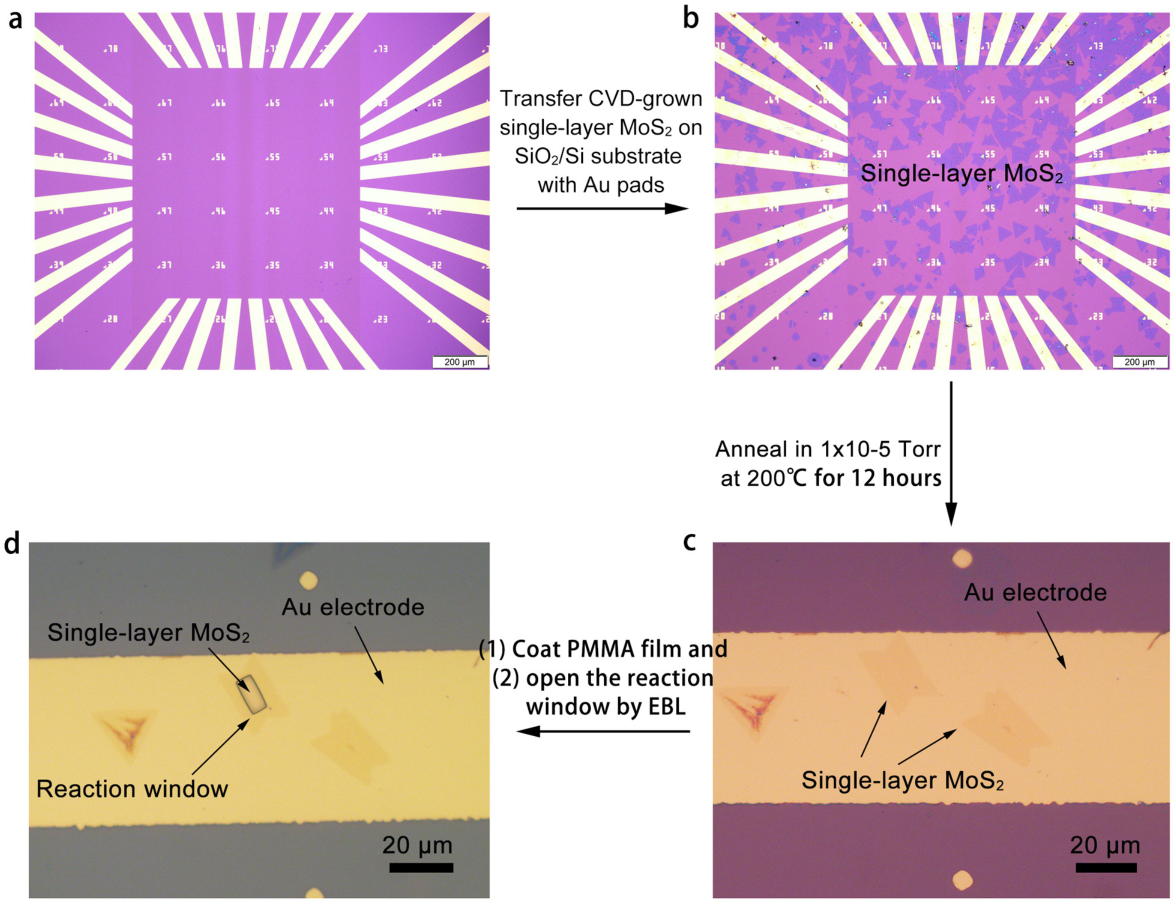

Supplementary Figure 17. Fabrication procedure for the single-layer MoS_2 microelectrode on Au substrate. **a**, SiO_2/Si substrates coated with Au pads. **b**, MoS_2/MoS_2-$MoSe_2$ film was transferred onto the substrate. **c**, 1-μm-thick PMMA was coated on the chip. **d**, A reaction window was opened to expose the region of interest on the film by using e-beam lithography.

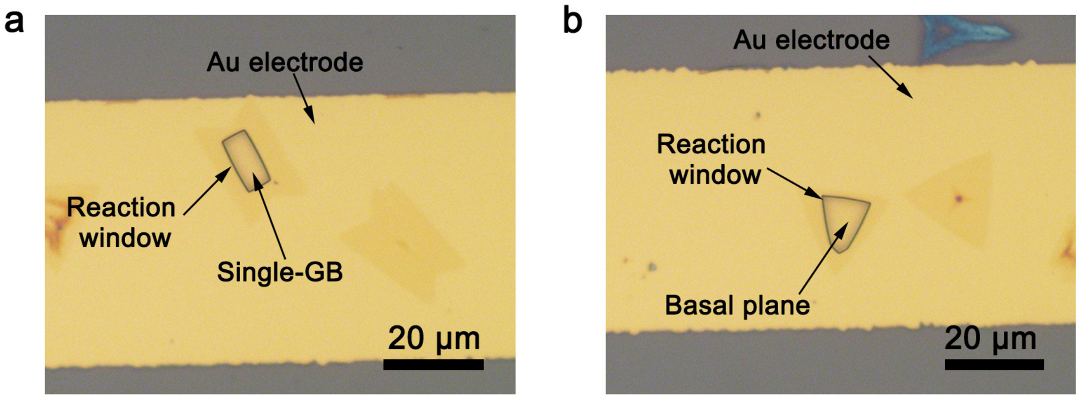

Supplementary Figure 18. Optical images of pure MoS_2 micro-electrochemical devices. The reaction windows are opened on single-GB (**a**) and basal plane (**b**) for HER measurement**.**

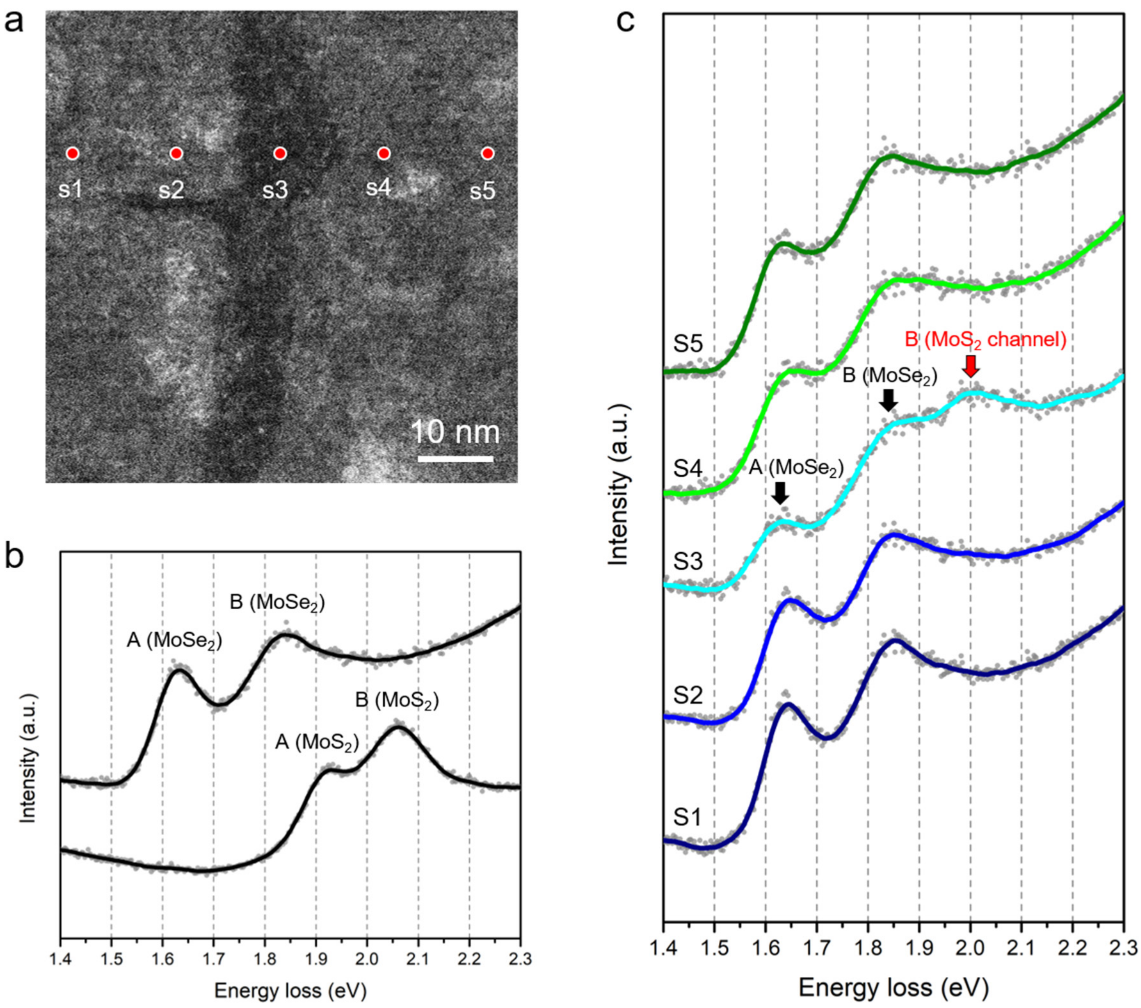

Supplementary Figure 19. Excitonic absorption spectroscopy of MoS_2 nano-channels. a, ADF-STEM image of a MoS_2 nano-channel. **b**, The low-loss electron energy loss spectroscopy (LL-EELS) spectra of pure $MoSe_2$ and MoS_2. **c**, Five LL-EELS spectra integrated at different positions highlighted in **a**. In this figure, the B exciton at 1.99 eV can be obtained of MoS_2 nano-channel (highlighted in **c**), which has 70 meV redshift compared with that of pure MoS_2 (**b**), indicating the strain-induced bandgap engineering within 10 nm scale.

Supplementary Tables

Parameter	Symbol	Value
GB energy (C_6 term)	σ_1	1 eV/Å
GB energy (C_3 term)	σ_2	0.5 eV/Å
Characteristic length	l	50 nm
Interface thickness	η	71
Coupling coefficient	λ	20
Desorption time	$\tilde{\tau}_s$	2.5
Diffusivity	$\widetilde{D}$	150
Mobility	μ_0	2
Interface tension anisotropy	δ	0.1
Equilibrated ξ	ξ_{eq2}	0.1

Supplementary Table 1. List of parameters for the phase-field simulation. Tildes are omitted for dimensionless parameters.
