# Supplementary material for: Strain-driven growth of ultra-long two-dimensional nano-channels
Source: Nat Commun. 2020 Feb 7;11:772. doi: 10.1038/s41467-020-14521-8 (PMC7005715; doi:10.1038/s41467-020-14521-8)
Supplement: Supplementary file 3 — Description of Additional Supplementary Files [file 41467_2020_14521_MOESM3_ESM.pdf]

# Description of Additional Supplementary Files

File Name: Supplementary Movie 1
Description: Phase filed simulation of the growth of 6-pont star $MoSe_2$.
